# The Accuracy of Intraoral Scanners in Maxillary Defects with Different Model Variations †

**DOI:** 10.3390/diagnostics14212368

**Published:** 2024-10-24

**Authors:** Sema Murat, Burcu Batak, Özge Aydoğ, Caner Öztürk

**Affiliations:** 1 Department of Prosthodontics, Faculty of Dentistry, Ankara University, Ankara 06560, Turkey; smurat@ankara.edu.tr; 2 Dentoper Fethiye-Private Clinic, Muğla 48300, Turkey; ozgeaydog@gmail.com; 3 Department of Prosthodontics, Faculty of Dentistry, Medipol University, Ankara 06570, Turkey; caner_ozturk86@hotmail.com

**Keywords:** maxillary defect, intraoral scanner, trueness, precision

## Abstract

**Background:** Advances in digital technology and intraoral scanners (IOSs) have the potential to enable accurate digital impressions for patients with maxillary defects. This study aimed to compare the accuracy of IOSs in completely and partially edentulous models with maxillary defects. **Methods:** Three polyurethane models—one completely edentulous (CE) and two partially edentulous, following Aramany classifications I (ACI) and II (ACII)—were created using stereolithography. These models were scanned with a desktop scanner to create reference models. Ten scans were performed using three different intraoral scanners (TRIOS 3, Primescan, and Virtuo Vivo). The IOS datasets were analyzed to assess trueness and precision using a two-way ANOVA and multiple-comparison tests with Bonferroni corrections (α = 0.05). **Results:** Both the model type and the IOS significantly influenced trueness and precision. The interaction between the model type and the IOS was found to be statistically significant (trueness: *p* = 0.001; precision: *p* = 0.005). The highest trueness was observed in the ACII model scanned with TRIOS 3 and Primescan. TRIOS 3 and Primescan also exhibited the highest precision in the ACII model. For Virtuo Vivo, there were no significant differences among the models (*p* = 0.48). **Conclusions:** Although intraoral scanners (IOSs) demonstrated significant differences in trueness when used in completely and partially edentulous models with maxillary defects, these differences may be considered clinically insignificant.

## 1. Introduction

Nowadays, the number of patients requiring obturator prostheses, which are used to regulate the disrupted oral–nasal relationship, prevent fluid leakage into the nasal cavity, and improve the patient’s vital functions in cases of maxillary defects, is steadily increasing. The fabrication of obturator prostheses involves challenging clinical procedures for both the clinician and the patient. Additionally, functional problems that arise after the prostheses are delivered can negatively impact patients’ quality of life [1].

The most critical factor in the success of an obturator prosthesis is the accurate capture of the maxillary anatomy, including the remaining teeth, soft and hard tissues, and defect boundaries. The detailed impression is crucial for closing the defect and improving the retention, stability, and functionality of the obturator prosthesis [2].

In recent years, intraoral scanners and medical imaging systems, such as computed tomography (CT) and cone-beam computed tomography (CBCT), have been employed as alternatives to conventional impression techniques in the fabrication of obturator prostheses [3,4].

Intraoral optical impression systems were designed to capture digital impressions of teeth, implants, and the surrounding soft tissues by generating three-dimensional (3D) datasets. The incorporation of digital impressions into clinical practice for the fabrication of obturator prostheses for patients with maxillary defects has eliminated certain problems associated with conventional impression techniques, such as the distortion of the impression, the risk of aspiration, and tearing or detachment of the impression material while being taken out of the maxillary defect [3,5,6]. Furthermore, the use of digital impressions as an alternative to conventional impressions decreases the number of impression steps required to construct the obturator prosthesis while shortening the duration of the treatment in total [1,2,3,4].

Some recent in vitro studies [3,4,5,7,8,9,10,11,12,13,14] focusing on the feasibility and accuracy of digitizing partially or completely edentulous arches with maxillary defects by using an intraoral scanner (IOS) have reported that the use of digital scans in maxillary defects could be considered in order to obtain more effective and accurate results when compared to conventional impression technique. However, capturing a detailed view of the real configuration of defect anatomy by using intraoral scanners (IOSs) can be challenging due to factors such as the size and location of the defects, as well as the presence of deep cavities or undercuts in the defect areas. Moreover, static images of soft tissues without border molding do not adequately capture crucial information related to frenulum and muscle movements during the fabrication of an obturator prosthesis.

In clinical practice, CBCT systems produce radiographic images instead of relying on reflective imaging, and they also have the potential to be employed in creating virtual impressions, which are a crucial first step in the process of obturator prosthesis reconstruction. The drawbacks of dental CBCT include issues such as scatter radiation, a restricted dynamic range, limited detail in soft tissue imaging, and artifacts caused by beam hardening due to dental materials and implants. In contrast, intraoral scanners offer the advantage of producing surface profiles of soft tissue, teeth, and restorations without the problem of scatter [15,16]. Therefore, intraoral scanners are the preferred method of choice for the accurate and detailed visualization of maxillary defect boundaries, adjacent teeth, and related dental restorations.

Nowadays, modern software technologies allow for the integration of digital images captured by intraoral scanners with CBCT image datasets to create three-dimensional (3D) virtual casts. These virtual casts can include the remaining dentition, defect areas, and both soft and hard tissues of patients with maxillary defects. The use of digital data generated by merging IOS images of the maxillary structures with a CBCT dataset of the defect’s anatomy is the most useful method to overcome the limitations of IOSs in the rehabilitation of patients with maxillary defects [3,4,7]. In particular, in cases of small, deep, and posterior defects, intraoral scanners are recommended to be used in combination with CBCT images due to their lower imaging accuracy in these areas [4,7].

Recently, Murat et al. [7] described a protocol that involves merging CBCT image data with intraoral optical scans of the remaining maxillary tissues in a partially edentulous patient with a maxillary defect to create an obturator prosthesis without the need for a conventional impression. Utilizing 3D printing, they produced a definitive cast with complex anatomy in a relatively short time for the fabrication of the obturator prosthesis. This 3D-printed definitive cast was then integrated into a conventional laboratory workflow to complete the fabrication of the obturator prosthesis [7]. The most important factor in the success of the merging technique is the accurate recording of oral tissues using intraoral scanners.

The term accuracy refers to how closely the measured dimensions of an object match its actual dimensions, and it is composed of trueness and precision parameters. Trueness is the degree to which a measurement aligns with the true size of the object, while precision refers to the consistency of repeated measurements of the same object. High trueness indicates that the result is close to the actual object, and high precision is achieved when measurements are predictable and repeatable [17]. Current intraoral scanners (IOSs) utilize different mechanisms to capture raw data in the form of point clouds, and the use of IOSs with varying scanning mechanisms, such as confocal microscopy, ultrafast optical scanning, optical triangulation, dynamic depth scan, and multiscan imaging technologies, may lead to differences in accuracy [18,19,20,21,22]. Given the potential differences in accuracy among various clinical intraoral scanner (IOS) systems, the aim of this study was to analyze the accuracy of three different IOSs, each utilizing different scanning mechanisms, in models of completely and partially edentulous patients with maxillary defects.

The null hypotheses tested were as follows:

**H1.**  *There would be no significant differences in trueness among the three different IOSs when used in different Aramany classification models*.

**H2.**  *There would be no significant differences in precision among the three different IOSs when used in different Aramany classification models.*

## 2. Materials and Methods

One completely edentulous (CE) and two different partially edentulous (ACI, Aramany classification I; ACII, Aramany classification II) maxillectomy defect models were designed using a software program (Mimics Innovation Suite version 22.0; Materialise, Leuven, Belgium). The polyurethane models were then fabricated using a stereolithography (SLA) desktop 3D printer (Formlabs Form 2, Formlabs Inc., Somerville, MA, USA).

A laboratory scanner (inLab X5 scanner; Dentsply Sirona, Bensheim, Germany) was utilized to generate a virtual three-dimensional (3D) reference model by performing a master scan of each polyurethane model. Three different intraoral scanners (IOSs) with various scanning mechanisms were used to capture the scans: TRIOS 3 (3Shape, Copenhagen, Denmark) uses confocal microscopy and ultrafast optical scanning technology; Cerec Primescan (Cerec-Dentsply Sirona, Bensheim, Germany) employs a smart pixel sensor with the dynamic depth scanning technology; and Virtuo Vivo (Dentalwings, Montreal, QC, Canada) functions with a blue laser scanner and multiscan imaging technology. Ten consecutive scans were performed with each intraoral scanner (IOS) by an experienced operator (O.A.). Before each scan, the operator conducted calibrations, following the recommendations provided by the IOS manufacturer. The scan strategies used in this study were as follows:

For the TRIOS 3, the scan began with the occlusal surface and covered the entire arch. The operator then returned to scan the lingual surfaces, as recommended by the manufacturer. The acquisition was completed by scanning the buccal surfaces.

For the Primescan, the scan began by capturing the lingual surfaces. The operator then scanned the occlusal surfaces across the entire arch, moving back to the starting point, and completed the acquisition by scanning the buccal aspects.

For the Virtuo Vivo, since the manufacturer did not provide a recommended scanning strategy, the scan path used for the TRIOS 3 was adopted. This involved starting with the occlusal surface, covering the entire arch, followed by scanning the lingual surfaces and completing the acquisition by scanning the buccal surfaces.

In all IOSs, the scanner tips were then guided in a zigzag pattern to scan the mucosal region, including the palate and the maxillectomy defect [9,23]. For the edentulous model, the scans were performed in a zigzag pattern, moving from the non-defective side to the defective side.

**Figure 1:**
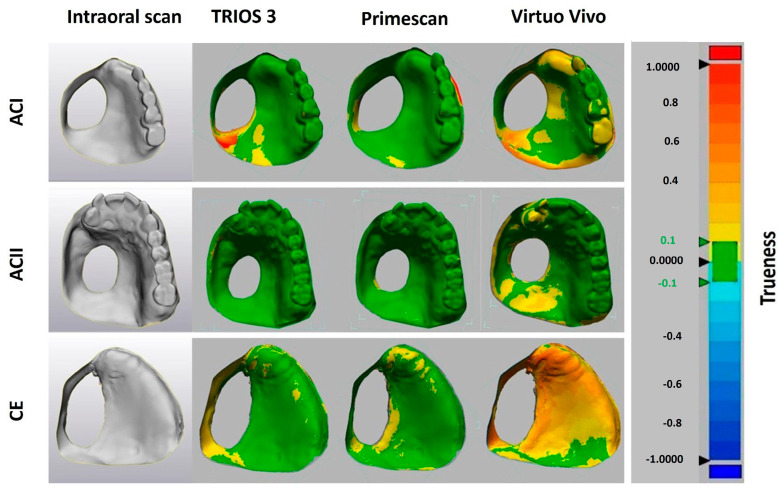
Geometrical comparison of the 3D deviations for assessment of trueness.

**Figure 2:**
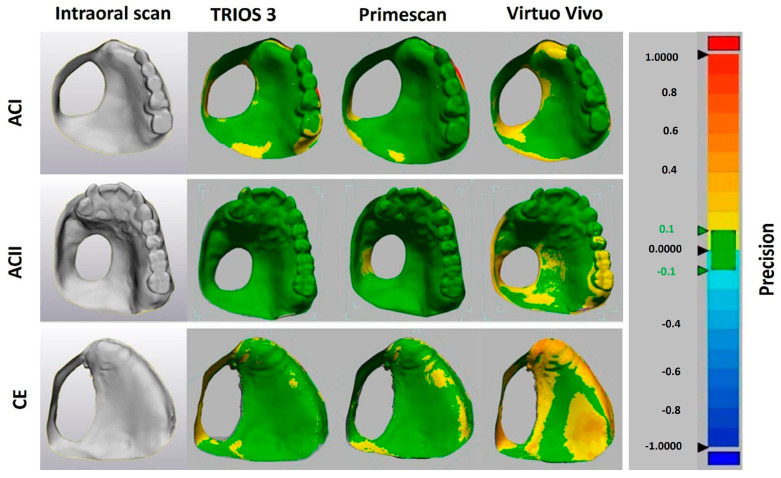
Geometrical comparison of the 3D deviations for assessment of precision.

The data files were converted into standard tessellation language (STL) files, which were then imported into 3D image processing software (Geomagic Control X 64; 3D Systems; Rock Hill, SC, USA) to assess the deviations of the 3D models obtained from the intraoral scanners (IOSs). Areas of the scan that extended beyond the clinically relevant peripheral border were cropped. The 3D datasets were then geometrically superimposed using the best-fit algorithm provided by the 3D evaluation software. The variation was calculated by cross-comparing different scans in each group (*n* = 10 for Trios 3, *n* = 10 for Primescan, and *n* = 10 for Virtuo Vivo for each model (ACI, ACII, and CE)) to assess precision (intergroup comparison), while the 3D differences between the intraoral scan data in each group (*n* = 10 for Trios 3, *n* = 10 for Primescan, and *n* = 10 for Virtuo Vivo for each model) and the reference data (master scan of each polyurethane model obtained using a laboratory scanner, *n* = 1 for each model) were considered to determine trueness (intragroup comparison) [24]. The software calculated the mean absolute 3D deviations using the root mean square (RMS) method. A value of 0 indicates perfect accuracy, while deviations in either direction from this value indicate inaccuracy (Figure 1 and Figure 2).

The data were analyzed using 2-way ANOVA. Multiple-comparison tests with Bonferroni corrections (α = 0.05) were used to determine the differences between the mean values (SPSS 19, SPSS, IBM, Chicago, IL, USA).

## 3. Results

**Figure 3:**
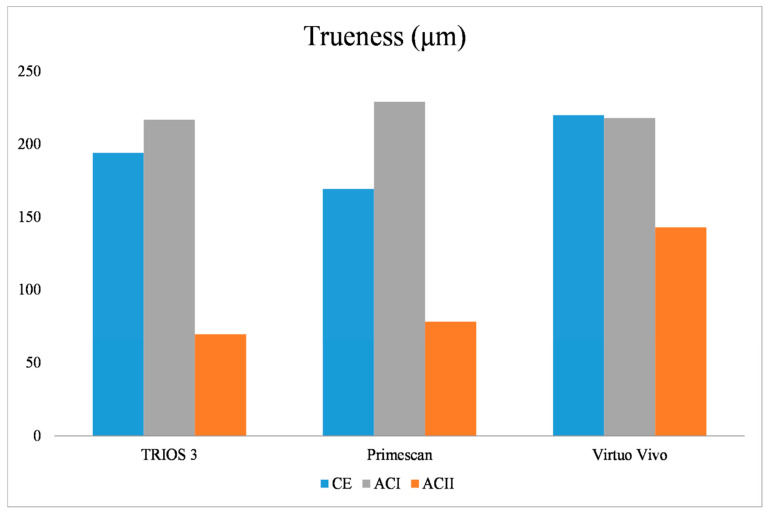
The 3D deviation values in terms of trueness for different scanner types among CE, ACI, and ACII models: CE: complete edentulous, ACI: Aramany Cl I, ACII: Aramany Cl II.

In terms of trueness, the type of model, the intraoral scanner used, and the interaction between the model type and the intraoral scanner were all found to be statistically significant (*p* = 0.001). For the ACI model, no significant differences were found among the IOSs (*p* = 0.54) (Table 1). However, for the CE model, there were significant differences among all IOSs (*p* = 0.001), with Primescan exhibiting the lowest 3D deviation and Virtuo Vivo the highest. In the ACII model, there were no significant differences between TRIOS 3 and Primescan (*p* = 0.57), but Virtuo Vivo showed the highest 3D deviation (*p* = 0.001) (Table 2). When analyzing TRIOS 3 across the different models, significant differences were found (*p* = 0.001), with ACI showing the highest 3D deviation and ACII the lowest. For Virtuo Vivo, ACII had the lowest 3D deviation, while no significant differences were found between ACI and CE (*p* = 0.99). Regarding Primescan, significant differences were noted among all models (*p* = 0.001), with ACI showing the highest 3D deviation and ACII the lowest (Figure 3).

**Figure 4:**
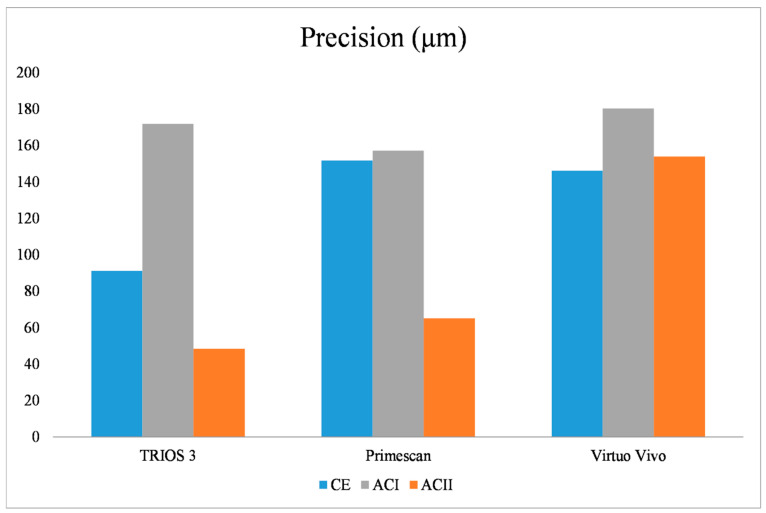
The 3D deviation values in terms of precision for different scanner types among CE, ACI, and ACII models: CE: complete edentulous, ACI: Aramany Cl I, ACII: Aramany Cl II.

In terms of precision, the effects of model type, intraoral scanner, and the interaction between model type and scanner were statistically significant (*p* = 0.005). For the CE and ACI models, no significant differences were observed among the intraoral scanners (*p* = 0.07 for CE, *p* = 0.307 for ACI) (Table 3). In the ACII model, there were no significant differences between TRIOS 3 and Primescan (*p* = 0.75), but Virtuo Vivo exhibited the highest 3D deviation (Table 4). When analyzing TRIOS 3 across different models, significant differences were found (*p* = 0.001), with ACI showing the highest 3D deviation and ACII the lowest. For Virtuo Vivo, there were no significant differences among the models (*p* = 0.48). Regarding Primescan, ACII had the lowest 3D deviation (*p* = 0.001), while no significant differences were found between ACI and CE (*p* = 0.95) (Figure 4).

## 4. Discussion

The first and second null hypotheses were rejected because statistically significant differences in trueness and precision were observed among the IOSs when used with different model variations with maxillary defects.

Some previous studies have attempted to evaluate the effectiveness of intraoral scanners for the digitization of completely and partially edentulous arches, prioritizing the use of digital impressions over conventional techniques [4,8,9,23,25,26,27,28,29]. These studies have shown that obtaining reliable digital scans of edentulous arches could be seriously challenging, even if possible. In fact, the accuracy of the models required for the fabrication of complete or partial removable dentures is generally lower than that required for fixed dentures [30]. Additionally, intraoral scanners capture the mucosal anatomy in a passive state, whereas conventional impression materials apply pressure to the oral mucosa, leading to mucosal displacement depending on the mucosa’s viscoelastic properties. The amount of mucosal displacement at the distal end of the denture base can reach up to approximately 300 µm, which is regarded as an indication of the clinical tolerance range for impression accuracy [31]. This tolerance range suggests that digitizing edentulous jaws using intraoral scanners (IOSs) can be a clinically acceptable alternative to conventional impression methods. Hayama et al. [32] demonstrated that the trueness of the mucosal area in digital impressions obtained using an intraoral scanner (Trophy Solutions, Carestream Health, Rochester, NY, USA) ranged from 54 to 180 µm, while the precision ranged from 109 to 215 µm. Consistent with the findings of Hayama et al. [32], the present study found that the trueness and precision values were within the range of mucosal displacement considered clinically acceptable.

Some recent studies have also indicated that conventional impressions of completely edentulous arches do not provide better accuracy compared to intraoral scanners (IOSs) [25,32]. In a recent in vitro study, Elbashti et al. [8] found that the average 3D deviations for the IOS in completely edentulous models with maxillary defects ranged from 168 µm to 170 µm, depending on the defect size. In contrast, the average 3D deviations for conventional impressions ranged from 197.2 ± 81.7 µm to 247.7 ± 128.8 µm, which were statistically higher than those of the IOS. They suggested that IOS images could be effectively used in completely edentulous patients with maxillary defects as a replacement for conventional impressions. In the present study, supporting the findings of Elbashti et al. [8], the mean 3D deviation values for the CE models were found to be between 169.2 and 219.9 µm, depending on the intraoral scanner used. Although the Primescan IOS demonstrated significantly higher trueness than TRIOS 3 and Virtuo Vivo for the CE model, all scanners showed a clinically acceptable error (less than 300 µm). No significant differences were found among the scanners in terms of precision for the CE model.

In another in vitro study, Elbashti et al. [9] examined the precision and trueness of digital impressions obtained using an intraoral scanner (3M True Definition, 3M ESPE) in maxillary defect models representing different Aramany classifications. They found that the Class II model, which includes fewer edentulous areas, exhibited the highest trueness compared to the Class I and Class IV models. Consistent with the findings of previous studies, they observed that the accuracy of the images decreases in the presence of large edentulous areas with smooth surfaces, due to the absence of reference points needed for aligning multiple images captured during the scanning process. Regarding precision, Elbasthi et al. [9] found that the Class II model exhibited lower precision compared to the Class IV model. They attributed this finding to the size and shape of the defect, noting that although the Class II model had more remaining teeth, it also featured a deeper and smaller defect that was more difficult for the scanner tip to reach compared to the Class IV defect. In the present study, while all scanners demonstrated the highest trueness for the Class II model, it was surprising to find that the trueness for the completely edentulous (CE) model was significantly higher than for the Class I defect model when using Primescan and TRIOS 3. This result might be explained by the deep undercut area on the buccal surfaces of the remaining teeth in the Class I defect model used in this study, which posed challenges in capturing accurate images of these regions. Furthermore, contrary to the findings of Elbasthi et al. [9], the present study showed statistically higher precision for the Class II defect model when using Primescan and TRIOS 3, whereas the precision for all scanners was not statistically different between the Class I and CE models.

The intraoral scanner (IOS) systems evaluated in this study enabled the rapid production of definitive digital 3D virtual casts that accurately represented the hard and soft tissue anatomy. This was achieved using various technologies: ultrafast optical sectioning and confocal microscopy (TRIOS 3), dynamic depth scanning technology (Primescan), and orthographic projection technology (Virtuo Vivo). In the present study, for the completely edentulous (CE) model, Primescan exhibited the lowest 3D deviation compared to TRIOS 3 and Virtuo Vivo. The superior accuracy of Primescan may be attributed to its dynamic depth scanning technology and associated software. Further in vivo and in vitro studies are needed to better understand the differences in accuracy across different scanners.

Ender and Mehl [33] demonstrated that the scan strategy or direction significantly affects trueness and precision. However, manufacturers do not provide specific recommendations on how to scan edentulous jaws. According to the literature [23,26,34], the most commonly preferred scan path for capturing surface data from edentulous jaws is a zigzag pattern. To ensure adequate overlap and accurate capture of adjacent structures, we adopted this zigzag scan path when imaging the models in the present study.

The main limitation of this study is that all scans were performed by a single experienced operator, which could influence the accuracy of the intraoral scans due to the operator’s level of expertise. Another limitation is that we did not consider various oral environment factors, such as the presence of saliva, temperature, humidity, patient movement, soft tissue movement, and the different light-reflective properties of hard and soft oral tissues due to the in vitro nature of the present study. These factors can significantly affect the accuracy of digital impressions. Therefore, further in vivo studies conducted with multiple operators are recommended to evaluate the effectiveness of various intraoral scanners (IOSs) in capturing digital impressions of partially or completely edentulous jaws with maxillary defects. In our notion, future studies that evaluate the accuracy of digital intraoral impressions in maxillary defects with dental implants, an aspect outside the scope of this study, should also be encouraged.

## 5. Conclusions

Within the limitations of this in vitro study, the following conclusions were drawn based on the findings:

In terms of trueness, the ACII model, which had more remaining teeth compared to the other models, demonstrated the highest trueness across all the IOSs tested.

In terms of precision, there were no significant differences among the IOSs for the CE and ACI models. However, for the ACII model, Virtuo Vivo showed the lowest precision.

Digitizing completely and partially edentulous models with maxillary defects using intraoral scanners appears to result in clinically acceptable errors (<300 µm) in terms of accuracy.

## Figures and Tables

**Table 1:** Analysis of variance of 3D deviations for trueness.

Model	Sum of Squares	df	Mean Square	F	Sig.
CE	0.013	2	0.006	18.171	0.001 *
ACI	0.001	2	0.000	0.625	0.54
ACII	0.032	2	0.016	43.601	0.001 *

* *p* < 0.05.

**Table 2:** Significant differences in trueness of various IOSs for all models.

Model	Scanner	Mean ± SD Trueness (μm)	Scanner	Mean ± SD Trueness (μm)	*p*
CE	Primescan	169.2 ± 19.4	TRIOS 3	194.1 ± 9.2	0.017 *
			Virtuo Vivo	219.9 ± 24.5	0.001 *
	TRIOS 3	194.1 ± 9.2	Primescan	169.2 ± 19.4	0.017 *
			Virtuo Vivo	219.9 ± 24.5	0.013 *
	Virtuo Vivo	219.9 ± 24.5	Primescan	169.2 ± 19.4	0.001 *
			TRIOS 3	194.1 ± 9.2	0.013 *
ACI	Primescan	229 ± 31.0	TRIOS 3	216.8 ± 17.0	0.58
			Virtuo Vivo	217.9 ± 30.6	0.63
	TRIOS 3	216.8 ± 17.0	Primescan	229 ± 31.0	0.58
			Virtuo Vivo	217.9 ± 30.6	0.10
	Virtuo Vivo	217.9 ± 30.6	Primescan	229 ± 31.0	0.63
			TRIOS 3	216.8 ± 17.0	0.10
ACII	Primescan	78.3 ± 16.0	TRIOS 3	69.5 ± 13.7	0.57
			Virtuo Vivo	142.9 ± 25.7	0.001 *
	TRIOS 3	69.5 ± 13.7	Primescan	78.3 ± 16.0	0.57
			Virtuo Vivo	142.9 ± 25.7	0.001 *
	Virtuo Vivo	142.9 ± 25.7	Primescan	78.3 ± 16.0	0.001 *
			TRIOS 3	69.5 ± 13.7	0.001 *

* *p* < 0.05.

**Table 3:** Analysis of variance of 3D deviations for precision.

Model	Sum of Squares	df	Mean Square	F	Sig.
CE	0.019	2	0.009	2.997	0.070
ACI	0.002	2	0.001	1.242	0.307
ACII	0.058	2	0.029	12.299	0.000 *

* *p* < 0.05.

**Table 4:** Significant differences in the precision of various IOSs for all models.

Model	Scanner	Mean ± SD Precision (μm)	Scanner	Mean ± SD Precision (μm)	*p*
CE	Primescan	151.7 ± 54.6	TRIOS 3	91.1 ± 41.9	0.09
			Virtuo Vivo	146.1 ± 66.4	0.98
	Primescan	151.7 ± 54.6	Primescan	151.7 ± 54.6	0.09
			Virtuo Vivo	146.1 ± 66.4	0.13
	Virtuo Vivo	146.1 ± 66.4	Primescan	151.7 ± 54.6	0.98
			TRIOS 3	91.1 ± 41.9	0.13
ACI	Primescan	157.1 ± 40.0	TRIOS 3	171.8 ± 28.6	0.59
			Virtuo Vivo	180.3 ± 24.1	0.28
	TRIOS 3	171.8 ± 28.6	Primescan	157.1 ± 40.0	0.59
			Virtuo Vivo	180.3 ± 24.1	0.84
	Virtuo Vivo	180.3 ± 24.1	Primescan	157.1 ± 40.0	0.28
			TRIOS 3	171.8 ± 28.6	0.84
ACII	Primescan	65.1 ± 14.43	TRIOS 3	48.4 ± 18.2	0.75
			Virtuo Vivo	153.9 ± 80.7	0.002 *
	TRIOS 3	48.4 ± 18.2	Primescan	65.1 ± 14.43	0.75
			Virtuo Vivo	153.9 ± 80.7	0.001 *
	Virtuo Vivo	153.9 ± 80.7	Primescan	65.1 ± 14.43	0.002 *
			TRIOS 3	48.4 ± 18.2	0.001 *

* *p* < 0.05.

## Data Availability

Dataset available on request from the authors. The raw data supporting the conclusions of this article will be made available by the authors on request.
